# Optimized holographic femtosecond laser patterning method towards rapid integration of high-quality functional devices in microchannels

**DOI:** 10.1038/srep33281

**Published:** 2016-09-13

**Authors:** Chenchu Zhang, Yanlei Hu, Wenqiang Du, Peichao Wu, Shenglong Rao, Ze Cai, Zhaoxin Lao, Bing Xu, Jincheng Ni, Jiawen Li, Gang Zhao, Dong Wu, Jiaru Chu, Koji Sugioka

**Affiliations:** 1 CAS Key Laboratory of Mechanical Behavior and Design of Materials, Department of Precision Machinery and Precision Instrumentation, University of Science and Technology of China, Hefei 230026, China; 2 Laser Technology Laboratory, RIKEN, 2-1 Hirosawa, Wako, Saitama 351-0198, Japan

## Abstract

Rapid integration of high-quality functional devices in microchannels is in highly demand for miniature lab-on-a-chip applications. This paper demonstrates the embellishment of existing microfluidic devices with integrated micropatterns via femtosecond laser MRAF-based holographic patterning (MHP) microfabrication, which proves two-photon polymerization (TPP) based on spatial light modulator (SLM) to be a rapid and powerful technology for chip functionalization. Optimized mixed region amplitude freedom (MRAF) algorithm has been used to generate high-quality shaped focus field. Base on the optimized parameters, a single-exposure approach is developed to fabricate 200 × 200 μm microstructure arrays in less than 240 ms. Moreover, microtraps, QR code and letters are integrated into a microdevice by the advanced method for particles capture and device identification. These results indicate that such a holographic laser embellishment of microfluidic devices is simple, flexible and easy to access, which has great potential in lab-on-a-chip applications of biological culture, chemical analyses and optofluidic devices.

In the past several decades, integrated microfluidic systems have been increasingly attracting interest due to their broad applications in chemistry, physics, biology and medicine^1^,^2^,^3^,^4^. Compared with macroscopic settings, microfluidic devices have distinct properties, such as low Reynolds numbers, small dimensions and tiny fluid volumes^5^,^6^,^7^. Recent research trends to integrate microdevices into microfluidic chips towards higher functionalization. Conventional fabrication methods, including mask-based and maskless lithography, have been successfully applied to fabricate functional microfluidic chips. For example, mask-based lithography, such as ultraviolet (UV) lithography^8^,^9^, Soft X-ray lithography^10^,^11^ and nanoimprinting lithography (NIL)^12^,^13^, have been used for fabrication of cell sorting devices, nano channels and optofluidic devices. However, the mask-based lithography greatly relies on additional mask or mold, which requires multi-step fabrication process. Compared with mask-based lithography, maskless lithography such as electron beam lithography (EBL)^14^, femtosecond laser direct writing^15^,^16^, focused ion beam (FIB)^17^ and femtosecond laser induced photodynamic assembly of nanoparticles^18^ is more flexible and appropriate for fabricating devices with customized applications, e.g., DNA manipulation and microfluidic mixture. However, their fabrication efficiency is low due to the point-to-point scanning strategy. Therefore, it is highly desirable to develop a simple, rapid and maskless processing technology capable of integrating functional structures into microchips.

Femtosecond laser microfabrication by two-photon polymerization (TPP) is a promising method to reach this end due to its unique advantages of programmable designability, high spatial resolution, and diversity of usable materials^19^,^20^,^21^,^22^. TPP has been used to integrate a variety of microstructures including overpass for guiding different fluids^23^, micromixer for high efficiency mixing of different fluids^24^, microfilter for controllable filtering of particles^25^ and center-pass optofluidic microlens array for 100% cell counting^26^ into microfluidic channels. However, due to the long processing time of the single point writing scheme^27^, TPP is limited to be used for laboratory investigation and prototype fabrication. To overcome the obstacle, the multipoint parallel scanning has been realized by applying SLM^21^, which splits the light into 3 × 3 or more parallel beams to significantly reduce the processing time by a factor of 1/9 or shorter. But parallel fabrication can only process array structures as all foci follow the same scanning route^28^. Moreover, the time-consuming issue during TPP integration is still serious in processing microstructures in a large field, for example, covering a millimeter-scale channel with functional microstructures. Recent studies have shown that patterned femtosecond laser beam shaping can provide higher efficiency in TPP fabrication^29^,^30^,^31^. When applying patterned beam shaping, there is no need to scan the focus, enabling high-speed processing by a single exposure. However, the resolution and surface quality were degenerated due to the unoptimized beam shaping technique. Previous beam shaping techniques are based on digital micromirror device (DMD) or phase modulated liquid crystal spatial light modulator (LC-SLM) to modify the amplitude or phase of laser. DMD generates patterned focus by adjusting the reflect angles of irradiated beams at each pixel^32^. The pixelated micromirror units switched between static black (‘off’) and white (‘on’) states. Thus, when using a DMD, spatial flat-top femtosecond laser is required to generate the same energy of beams reflected by each pixel, otherwise the central intensity will be larger than that of edge when Gaussian beam is applied. When using a phase modulated LC-SLM, computer generate hologram (CGH) is loaded on LC-SLM to generate desired shaped focus^33^. But the shaped focus suffer from speckle noise caused by the randomness of the phase angles, which would result in flawed microstructures^34^,^35^. To suppress the speckle noise, several approaches such as time-average^19^ and shift-average^36^ have been used to improve the quality of focus. These “average” approaches calculate tens of CGHs containing the same target intensity and random speckle noise. Thus, the noise can be averaged by successively loading CGHs on LC-SLM. However, loading tens of CGH on LC-SLM will cost hundreds of milliseconds and results in throughput bottleneck.

In this paper, we apply a phase modulated LC-SLM to generate shaped patterns with highly spatially uniform energy by adopting a mixed region amplitude freedom (MRAF) algorithm^37^, which has been put forward to realize smooth patterned focus for atom trapping. Utilizing the smooth patterned focus generated by MRAF algorithm, we develop a technique termed MRAF-based holographic patterning (MHP) method, to realize rapid integration of microfluidic device with high resolution and high surface quality. Firstly, the rectangular light field distribution along the principal optical axis is theoretically simulated by MRAF, whose uniformity is higher than that derived from Gerchburg-Saxton (GS) algorithm. This is further verified by fabricating rectangle patterns with MRAF-calculated computer generated holograms (CGHs) in our experiment. Secondly, numbers, letters, Olympic logo and Quick Response (QR) code are fabricated by fixed point exposure of several pulses, which demonstrates this approach is capable of rapidly fabricating arbitrary structures with good quality. Thirdly, microtrap structures, QR code and letters are integrated in a channel. The trap structures covering the whole bottom of the 5000 μm × 170 μm channel is completed within 10 seconds. The QR code fabricated with less than 10 pulses can be easily recognized by a commercial modern smartphone, which can be used for device identification. Finally, the “lab on chip” microdevice integrated with the trap structures is tested with SiO_2_ particles suspension with diameters of 3.0 μm, 5.0 μm and 10.0 μm in alcohol solution, and the results show that 10.0 μm beads can be completely captured while other two size beads pass successfully.

## Methods

### Calculation process of the MRAF-CGH

A variety of iterative Fourier transform algorithms (IFTAs) like Gerchburg-Saxton (GS) and Adaptive-Additive (AA) algorithm are widely used to minimize the difference between the desired intensity distribution and that generated by CGH at the output plane^38^,^39^. Compared with GS and AA algorithms, MRAF algorithm can obviously improve accuracy. Their basic steps follow the frame of IFTA except for two factors: the initial phase chosen in iteration and the way to generate new light field at the output plane. Unlike GS algorithm choosing random phase as the initial phase, MRAF optimizes it to ensure that the output plane does not contain optical vortices, points characterized by a phase singularity and zero intensity, since an IFTA is not able to eliminate it^34^,^35^. After choosing the initial phase of algorithm, IFTA calculates the light field at the output plane by a fast Fourier transform of the chosen phase combined with incident light amplitude. Then the amplitude of the output plane is replaced by the desired intensity distribution in GS algorithm. In contrast, the MRAF algorithm defines the amplitude of output field as a mixture of the target and former output field. As MRAF and GS follow the same iteration step, their calculation time is almost the same. The calculation details are shown in Supplementary Fig. S1.

### High-efficiency fabrication of microtraps by MRAF-based holographic femtosecond laser processing method

**Figure 1:**
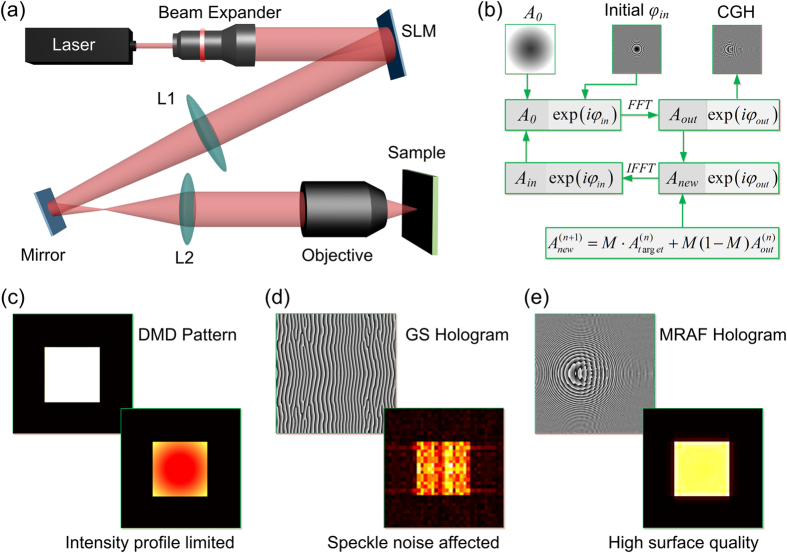
Fabrication setup and CGH calculation of MHP method. Schematics of intensity distribution by DMD, GS-CGH and MRAF-CGH based single exposure approach. (**a**) Schematic diagram of experiment setup of the MHP approach based on SLM. The SLM is placed at the back focal plane of L1. The focal length of L1 is 600 mm and that of L2 is 200 mm. L1, L2 and objective are confocal. (**b**) Flow-chart diagram of MRAF algorithm. The differences between MRAF and GS algorithms are “initial phase” and “new field in output plane”. (**c**) Single-exposure laser direct patterning by DMD. The optical intensity distribution at the focal plane is Gaussian due to the profile of incident Gaussian beam. (**d**) Single-exposure holographic femtosecond laser direct patterning by GS algorithm. The optical intensity distribution at the defocused plane can be designed as a complex rectangle by GS algorithm calculated CGH. (**e**) Single-exposure holographic femtosecond laser direct patterning by MRAF algorithm. Compared with GS algorithm, the optical intensity distribution at the focal plane is significantly improved.

Figure 1a shows a schematic diagram of experimental setup for femtosecond laser MHP fabrication. The laser source is a regenerative amplified Ti:Sapphire (Legend Elite-1 K-HE) at central wavelength of 800 nm with pulse duration of 104 fs and repetition rate of 1 kHz. After passing through a beam collimation system and a 4f configuration with a magnification of 0.3, femtosecond laser pulses irradiate onto a reflective liquid crystal on silicon (Holoeye, Pluto NIR-2, resolution of 1920 pixel × 1080 pixel, pixel pitch of 8 μm, and diagonal of 0.7 inch) for holographic processing. Designable CGH are encoded on the central 1080 × 1080 pixels to generate any desired patterns. The patterns are shifted from the center by loading blazed grating on SLM and a high-pass filter is placed at the focal plane of L1 to block the 0 order beam. A 100× microscope objective lens (Olympus, N.A. 0.9) is used to focus the phase-modulated femtosecond laser beam into the sample. The sample is prepared by drop casting a mixture of SZ2080^40^ and acetone (volume ratio 1:5) on a cover glass or in a channel.

### Fabrication of microchannel

The open microfluidic channel with 1 cm-length is fabricated by UV lithography. The glass is spin coated by SU-8 with 5 s slope to 1500 rpm and kept for 20 s. Designed channel can be obtained by UV exposure through a mask and subsequent development. In this work, the dimensions of the channels were designed to be 70–170 μm in width and 15 μm in depth.

## Results and Discussion

### Optimized MRAF algorithm for MHP fabrication

Before giving the mathematical details of MRAF algorithm, we briefly review the operation of an IFTA. IFTA is widely used for generating the CGH which converts a light field at the input plane to a target intensity distribution at the focal plane. As the amplitudes of light at the input plane and output plane are fixed, the IFTA does not have an analytical solution. Figure 1b shows the block diagrams of IFTA algorithm. The initial phase 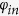 in MRAF is composed of linear and conical gradients to avoid introducing undesired optical vortices. After a fast Fourier transform (*FFT*) of the chosen phase combined with incident light amplitude, a new light field at the output plane is generated by replacing the amplitude with the desired intensity distribution in GS algorithm, which is given by 
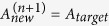
. In comparison, for the MRAF algorithm, the amplitude of output field is defined as 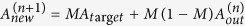, which is a mixture of the amplitude of target and former output plane according to the parameter *M*. Here *M* determines the rate of mixture of target and former output amplitude, which greatly influences the energy utilization rate and target uniformity of CGH. Besides, the new output field only replaces the amplitude in signal region, while the noise region keeps the former amplitude into the next iteration. The signal region is a ring-shape in this work and set large enough to contain the target pattern, and the noise region is the entire plane out of signal region. After calculating the merit 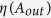 of the output plane, when 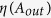 does not improve with repeated iterations, the phase of the input plane will be output to generate the CGH, otherwise they will keep the phase information and replace the amplitude by the incident light to start another circulation loop until the figure of merit stagnates.

### Theoretical and experimental comparison of MRAF and GS algorithms

In patterned TPP fabrication, the spatial intensity distribution is extremely important for getting a better surface quality. GS algorithm, as one of the most famous algorithms and the origin of MRAF algorithm, is not suitable for generating continuous light distribution. Figure 1c–e show the schematic diagram of femtosecond laser single exposure based on DMD, GS-CGH and MRAF-CGH. In DMD-based approach, incident light is modified into a patterned continuous distribution to create the structure by a single exposure. This method can significantly reduce the fabrication time, but the intensity inhomogeneity ascribed to Gaussian beam results in deterioration of patterned structures. In comparison, the GS-CGH based single exposure, shown in Fig. 1d, can get a continuous pattern by coding the phase of incident beam. However, this method is suffered from low signal to noise ratio (SNR), which will lead to a decrease in surface quality. As the noise region of MRAF-CGH is out of our interest, the SNR of MRAF-CGH is measured in the signal region instead of the whole focal plane. The SNR is calculated by 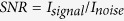. Here, *I*_*signal*_ is the target intensity, *I*_*noise*_ = *I*_*total*_ − *I*_*signal*_. The MRAF-CGH based single exposure keeps the advantages of DMD based approach but with no need of flat-top energy distribution of incident beam. As shown in Fig. 1e, a MRAF-CGH has high SNR to ensure both high speed and good surface quality of the achieved structures.

**Figure 2:**
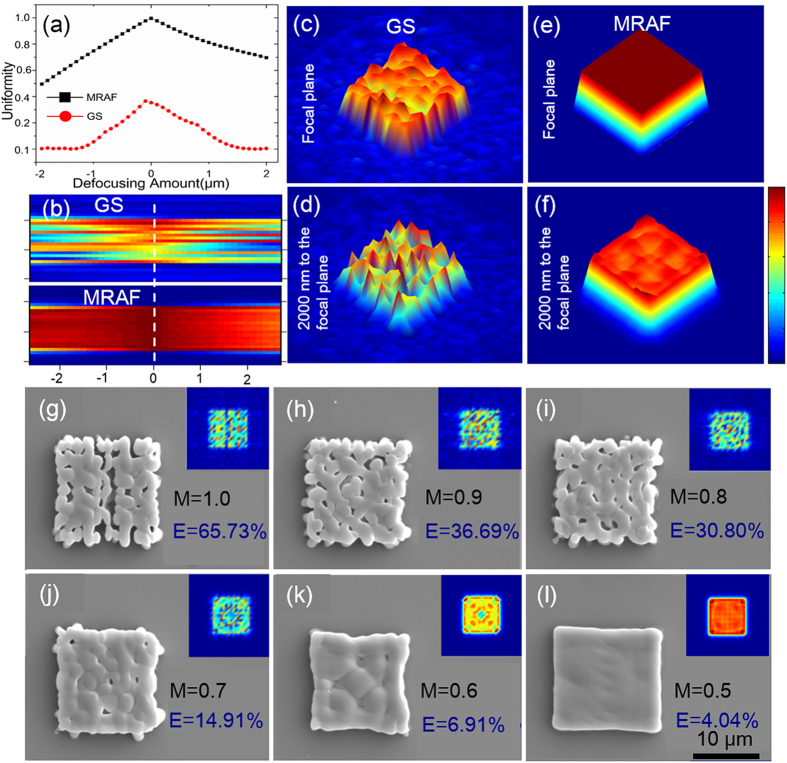
Simulation and optimization of MRAF-CGH for MHP fabrication. (**a**) The plot of the uniformity of MRAF (black rectangles) and GS (red circles) algorithms as a function of defocusing value. (**b**) The intensity distribution at the meridian plane. X axis is the defocusing value (μm). (**c**,**d**) The distributions at the focal plane and 2000 nm from the focal plane of CGH calculated by GS algorithm. (**e**,**f**) Those of CGH calculated by MRAF algorithm. (**g**–**l**) The top-view SEM images of rectangles fabricated by MRAF single-CGH-exposure approach with different M value from 1.0 to 0.5. The surface quality is obviously improved when the M value declines. E at the bottom of each structure is the energy utilization rate of each CGH. Scale bars are 10 μm.

Here, a simulation of intensity distribution by GS and MRAF algorithms is proposed based on Huygens-Fresnel diffractive integral,


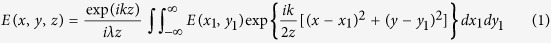


where *x, y* are the coordinates for initial field at the backside of the lens, while *x*_1_, *y*_1_ are the coordinates at the observation plane, *z* is the distance from SLM back surface to the observation plane, and *k* is the wavenumber. Figure 2a shows the uniformity in a sagittal plane with different defocusing values. The uniformity of MRAF algorithm is higher than that of GS algorithm. The focal plane gives the best uniformity for both. But it should be noted that the uniformity of GS is 36.62% and rapidly decreases to 10% at 2000 nm from the focal plane. This results in flawed and disjoint structures in TPP fabrication. In contrast, the uniformity of MRAF is nearly 100% at the focal plane and keeps upon 65% even at 2000 nm. The distribution at the meridian plane is presented in Fig. 2b to show the uniformity along the light axis visually. The simulated distribution of MRAF-CGH with M = 0.5 at the focal and defocused plane does not change too much as light spreads, which can be beneficial to achieve a better surface quality in TPP fabrication.

In order to avoid undesired flawed structures, the parameters of MRAF algorithm need to be optimized. Several CGHs with the same rectangle target were calculated to investigate the influence of parameters of MRAF on fabrication quality. The initial phases are all chosen as conical gradients to eliminate optical vortices. As we mentioned above, the ratio of target and former output field amplitude, which is called *M* value, influences the energy utilization rate of CGH and uniformity of laser intensity distribution. In simulation, when the *M* value increases, the laser utilization rate raises while the uniformity of laser intensity distribution decreases (Supplementary Fig. S2). The energy utilization rate of CGH with M = 1.0 is 65.73% and reduces to 4.04% when *M* = 0.5. This indicates the laser utilization efficiency is sacrificed in order to get higher surface quality. As we know, TPP takes place when the exposure energy exceeds the polymerization threshold of photoresist. So *M* should be optimized in order to get higher surface quality and at the same time to keep the laser density exceed the TPP threshold. We find that it is hard to perform TPP in a 10 μm × 10 μm field by single exposure due to the insufficient energy utilization rate when *M* is smaller than 0.5. Thus, 6 CGHs containing the phase information of the same rectangle pattern were calculated with different *M* values from 0.5 to 1.0. The MRAF-CGH is similar to GS-CGH when *M* = 1. Experimentally, six rectangles were fabricated by these 6 CGHs with the same exposure time (5 ms) and different pulse energy (0.6–20 μJ) [Fig. 2g–l], showing the surface quality is obviously improved as the *M* value decreases. The pulse energy varies to maintain the exposed energy of signal region is the same. The exposure time and energy was chosen according to the experimental results in Supplementary Figs S3 and S4. The energy was measured only in the signal region. We can find that *M* = 0.5 can ensure not only high surface quality but also sufficient polymerization in fabricating microstructure in spite of the energy utilization rate of 4.04%.

### Single-exposure TPP fabrication of high quality 2D microstructures on flat glass surface

**Figure 3:**
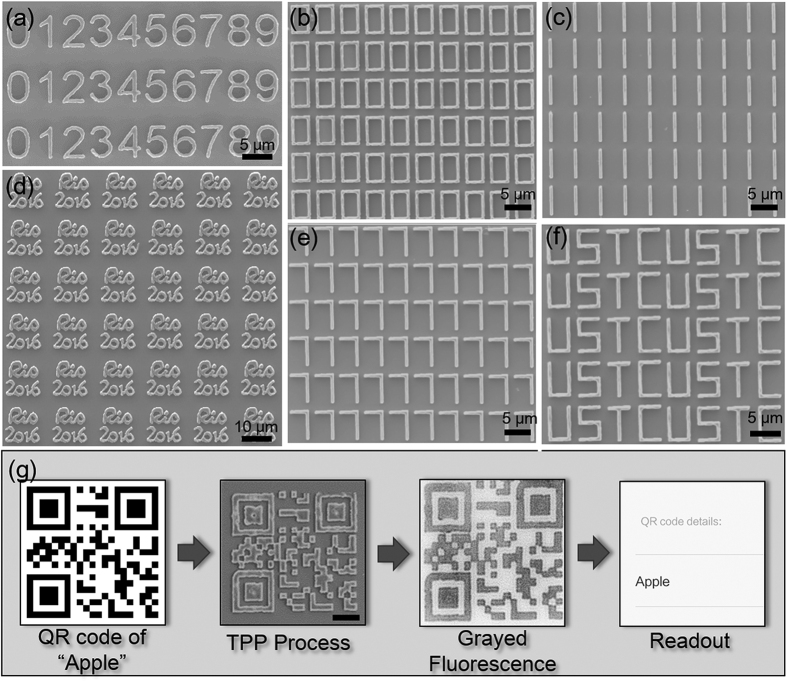
TPP fabrication of micropatterns on flat glass surface by the optimized MRAF-CGH. (**a**–**f**) SEM images of numbers (serial, “0”, “1”, “7”), letters (“USTC”) and Olympic logo. The fabrication time of each array is within 400 ms. (**g**) The reading procedure of the TPP fabricated QR code “Apple”. The fluorescence image of QR code was achieved by a fluorescence microscopy and further grayed and reversed to be read by a handle reorganization device. Scale bars are 10 μm.

Owing to the high SNR of MRAF-CGH, a single MRAF-CGH can produce a patterned focus with low influence of speckle noise, which makes MHP approach possible for integration of microdevices in microfluidics devices. In order to show the capability of the MHP approach in embellishing micro-fluidic chips, patterns and numbers were firstly fabricated on a flat cover glass. As shown in Fig. 3, a variety of high quality micropatterns including numbers, letters and Rio Olympic logo were realized with several laser pulses. Although the patterns look simple, they include curve (in Fig. 3a), right angle (Fig. 3b,e,f), line (Fig. 3c) and complex combination of them (Fig. 3d). The spatial resolution of the structures could be better than submicrometer [Fig. S5]. Each microstructure was formed by several pulse exposure of 20 μJ, which corresponded to the fabrication times as short as 5 ms in Fig. 3a–c,e,f, and 10 ms in Fig. 3d for 1 kHz repetition rate of ultra-high energy density amplifier laser source. The pulse energy was measured before the modulation. In this way, the arrays of microstructures can be realized in the field of 200 μm × 200 μm in several hundred milliseconds by the area-by-area expose of patterned focus beam. In addition, full coverage on a 10 mm × 1 mm cover glass with various micro structures can be achieved in only 1 minute, which is very close to the time cost by UV lithography. More importantly, this technique does not need a prefabricated mask and does offer higher flexibility and resolution. Furthermore, a 21 × 21 pixels QR code was fabricated to demonstrate the fabrication quality of single CGH approach. The QR code was separated into 3 × 3 blocks and each block was realized with 10 pulses of 20 μJ. The readability of created QR code was then proved by a readout test, shown in Fig. 3g. Firstly, the fluorescence image of QR code was obtained by a fluorescence microscope. Then, the fluorescence was grayed and reversed for the further identify test. Finally, the encoded data was decoded by a modern smartphone. The test results show that the fabricated QR code can be easily identified by a handle device, verifying a commonly used detector is available to read the micro QR code achieved by the MHP approach. By this means, the information of a microfuidic devices can be fast encoded in the microstructures and recognized when needed. The detail of reading process is shown in Supplementary Fig. S6 and Video1.

### Rapid integration of microtrap array and identification pattern of microdevice through single-exposure fabrication

The size-selective purification of microscopic objects is essential for a broad spectrum of applications in bio-analytics, clinical medicine and bead-based assays^41^,^42^,^43^,^44^. It has a variety of applications in the calculation of individual populations of cells from heterogeneous samples or circulating tumor cells (CTCs), and platelet separation from blood^45^. As a proof-of-concept demonstration of the efficiency and flexibility of the MHP approach, we attempted to integrate a simple but effective functional microdevice into the microfluidic chip for microparticles capture and separation. By taking advantage of the high flexibility and efficiency of our approach, microdevices with flexible designs can be fabricated in a very short time without prefabricated masks. Meanwhile, the quick identification of the microfluidic device based on a computer-recognizable micro-pattern implemented is increasingly demanded for batch processing. The MHP approach can rapidly integrate specific marks such as QR-codes in each device without any additional requirement in experimental setup and processing.

**Figure 4:**
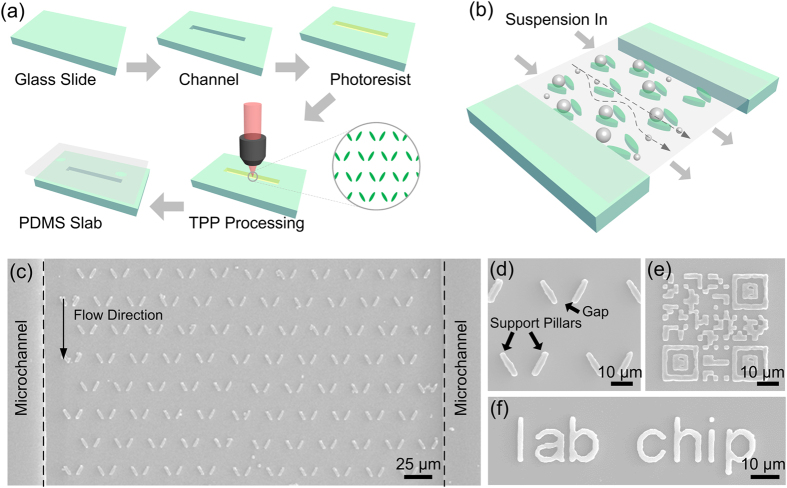
Rapid integration of microtraps and identification of microdevice in a microchannel. **(a)** The schematic diagrams of fabrication process (i) Preparation of micro-fluidic channel by UV lithography; (ii) surface coating with photoresist of SZ2080; (iii) single exposure fabrication of trap array and information of microdevice; (iv) covering of the device with PDMS slab. (**b**) The capture process. (**c**) The SEM image of integrated microdevice. (**d**–**f**) The details of traps, QR code and letters. Scale bars are 25 μm in (**c**), 10 μm in (**d**–**f**).

The schematic diagram of fabrication procedure is shown in Fig. 4a. SZ2080 was drop casted in the channel followed by a scrape process to remove polymer upper than the channel. The micro trap structures will capture particles which are larger than the gap size and allow smaller ones to pass through or move around, as shown in Fig. 4b. 1675 microtraps with 8 μm gap-size were fabricated (5 pulses with 15 μJ) in a 5000 × 170 μm channel by the MHP approach. Due to the high efficiency of the MHP approach, the total fabrication time was reduced to less than 10 seconds. Figure 4c is the SEM image of the “lab on a chip” device integrated with the traps, with its sign at the left bottom and a QR code at the right bottom. The QR code was fabricated in the same way as shown in Fig. 3g and could be also easily read by a handle device. The detailed SEM image of trap structure, QR code and letters are shown in Fig. 4d–f respectively, verifying the high quality of the MHP approach in processing chip device.

### Characterizing the function of microtrap by SiO_2_ microparticle

**Figure 5:**
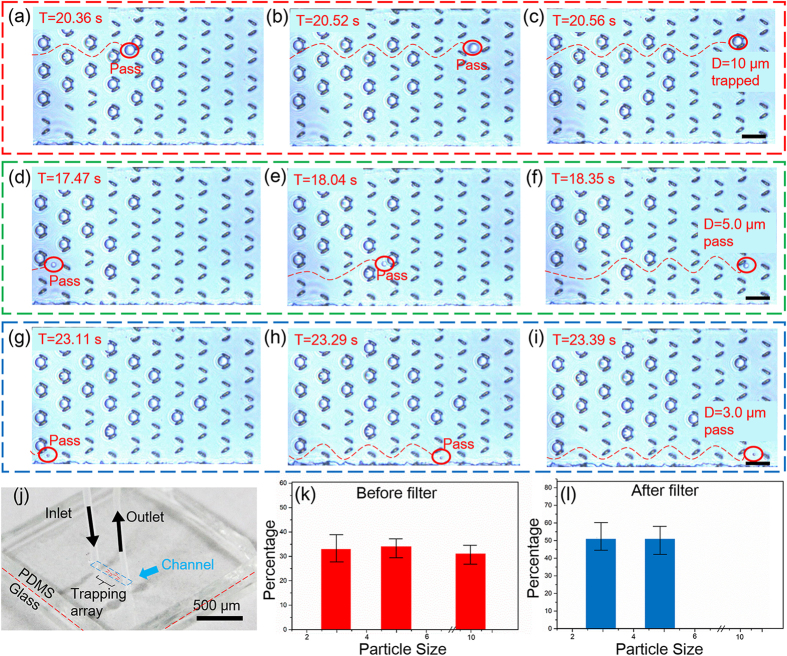
Trapping of big particles by the rapidly fabricated microtrap array. (**a**–**c**) A 10.0 μm SiO_2_ particle was captured by the microtraps. (**d**–**f**) A 5.0 μm SiO_2_ particle can easily pass the traps. (**g**–**i**) A 3.0 μm SiO_2_ particle can also easily pass the traps. (**j**) The photography of the microdevice. (**k,l**) Percentages of the microparticles for different sizes before and after the microtrap array. Scale bars are 30 μm.

To demonstrate the function of trap arrays with 8 μm gap, silica (SiO_2_) particles with diameters of 10.0 μm, 5.0 μm, and 3.0 μm mixed in alcohol solution were introduced into the integrated “lab on a chip” device. Thus, this device is expected to capture only the microparticles with the diameter of 10.0 μm. Please note that our technique can flexibly adjust the gap size of microtraps, which enables capturing various particles in terms of size. Figure 5a–i show time-lapsed optical microscopy images of flow of the mixture of three different-size particles in the trap array. It can be clearly identified that the particles smaller than the gap size can easily pass or flow around the traps. On the contrary, the biggest ones of 10.0 μm diameter are captured by the traps. As soon as a single trap structure successfully captures a particle, the trap is “blocked” to produce the streamline flow around the trap. The biggest particles move around the blocked trap until it is captured by another unblocked trap, while the smaller ones still pass or flow around traps, as shown with the red dashed lines in Fig. 5a–i. Statistic results (Fig. 5k,l) show that 100% of particles with size of 10.0 μm diameter have been successfully trapped. In contrast, particles smaller than 8 μm can completely pass through the trap array, presenting an excellent filtering and trapping capability [Supporting Video 2].

## Conclusions

In conclusion, MHP approach based on LC-SLM is found to be a powerful technology for functionalizing microfluidic devices. Optimized MRAF algorithm has been developed to generate high quality shaped beam for single-exposure patterned TPP. By using this method, a series of micropatterns including line, curve and complex structures were built in hundreds of milliseconds. Simple but effective microtrap arrays were fast integrated in a 5000 μm × 170 μm channel to demonstrate the ability in separating SiO_2_ particles. The MHP approach saves the processing time equivalently to the time cost by UV exposure method, while achieves much higher resolution and flexibility. Moreover, the flexibility of the MHP approach allows us to integrate chip information such as identification and QR codes in the channel without additional procedure. We believe that this technology will offer a flexible, facile and efficient way for “lab on a chip” device fabrication in terms of increase of integration degree and functionality in microfluidic chips, and thus will provide wider applications in chemical and biological communities. Currently, MHP approach is able to rapidly fabricate high quality 2D structures. Note that the light intensity distribution of MRAF along optical axis is not a Gaussian shape, making it hard to control the height of fabricated structures if 3D structures are desired. However, additional axial confinement technique such as spatiotemporal focusing^46^ can be applied combined with the proposed MRAF method to further improve the axial resolution and realize true 3D microstructures with a single exposure. In addition, the MHP can also be combined with point-to-point scanning TPP for rapidly fabricating 3D large-area functional microdevices and integrated microsystems.

## Additional Information

**How to cite this article**: Zhang, C. *et al*. Optimized holographic femtosecond laser patterning method towards rapid integration of high-quality functional devices in microchannels. *Sci. Rep.* **6**, 33281; doi: 10.1038/srep33281 (2016).

## Supplementary Material

Supplementary Information

Supplementary Video 1

Supplementary Video 2

## References

[b1] Whitesides G. M. The origins and the future of microfluidics. Nature 442, 368–373 (2006). 16871203 10.1038/nature05058

[b2] Yager P. . Microfluidic diagnostic technologies for global public health. Nature 442, 412–418 (2006). 16871209 10.1038/nature05064

[b3] Tia S. & Herr A. E. On-chip technologies for multidimensional separations. Lab Chip 9, 2524–2536 (2009). 19680577 10.1039/b900683b

[b4] Barbulovic-Nad I., Au S. H. & Wheeler A. R. A microfluidic platform for complete mammalian cell culture. Lab Chip 10, 1536–1542 (2010). 20393662 10.1039/c002147d

[b5] Craighead H. Future lab-on-a-chip technologies for interrogating individual molecules. Nature 442, 387–393 (2006). 16871206 10.1038/nature05061

[b6] Daw R. & Finkelstein J. Lab on a chip. Nature 442, 367–367 (2006).

[b7] Velve-Casquillas G., Le Berre M., Piel M. & Tran P. T. Microfluidic tools for cell biological research. Nano today 5, 28–47 (2010). 21152269 10.1016/j.nantod.2009.12.001 PMC2998071

[b8] Liu B. . Characterization of molecular transport in poly (dimethylsiloxane) microchannels for electrophoresis fabricated with synchrotron radiation-lithography and UV-photolithography. Lab Chip 4, 368–371 (2004). 15269806 10.1039/b315674e

[b9] Cox-Muranami W. A., Nelson E. L., Li G. & Bachman M. Large area magnetic micropallet arrays for cell colony sorting. Lab Chip 16, 172–181 (2016). 26606460 10.1039/c5lc01131k PMC6201277

[b10] Romanato F. . X-ray lithography for 3D microfluidic applications. Microelectronic Engineering 73, 870–875 (2004).

[b11] Falcaro P. . Fabrication of Advanced Functional Devices Combining Soft Chemistry with X‐ray Lithography in One Step. Adv Mater 21, 4932–4936 (2009). 25376948 10.1002/adma.200901561

[b12] Bilenberg B. . Topas-based lab-on-a-chip microsystems fabricated by thermal nanoimprint lithography. Journal of Vacuum Science & Technology B 23, 2944–2949 (2005).

[b13] Nilsson D., Balslev S. & Kristensen A. A microfluidic dye laser fabricated by nanoimprint lithography in a highly transparent and chemically resistant cyclo-olefin copolymer (COC). Journal of Micromechanics and Microengineering 15, 296 (2004).

[b14] Turner S., Perez A., Lopez A. & Craighead H. Monolithic nanofluid sieving structures for DNA manipulation. Journal of Vacuum Science & Technology B 16, 3835–3840 (1998).

[b15] Liao Y. . Rapid prototyping of three-dimensional microfluidic mixers in glass by femtosecond laser direct writing. Lab Chip 12, 746–749 (2012). 22231027 10.1039/c2lc21015k

[b16] Xu B. . Fabrication and multifunction integration of microfluidic chips by femtosecond laser direct writing. Lab Chip 13, 1677–1690 (2013). 23493958 10.1039/c3lc50160d

[b17] Campbell L., Wilkinson M., Manz A., Camilleri P. & Humphreys C. Electrophoretic manipulation of single DNA molecules in nanofabricated capillaries. Lab Chip 4, 225–229 (2004). 15159783 10.1039/b312592k

[b18] Wang H. . Photodynamic assembly of nanoparticles towards designable patterning. Nanoscale Horizons 1, 201–211 (2016). 10.1039/c5nh00065c 32260622

[b19] Zhang C. . An improved multi-exposure approach for high quality holographic femtosecond laser patterning. Appl Phys Lett 105, 221104 (2014).

[b20] Hu Y. . Laser printing hierarchical structures with the aid of controlled capillary-driven self-assembly. Proceedings of the National Academy of Sciences 112, 6876–6881 (2015). 10.1073/pnas.1503861112 PMC4460519 26038541

[b21] Xu B. . High efficiency integration of three-dimensional functional microdevices inside a microfluidic chip by using femtosecond laser multifoci parallel microfabrication. Sci Rep-Uk 6, 19989 (2016). 10.1038/srep19989 PMC4730193 26818119

[b22] Lao Z. . Capillary Force Driven Self-Assembly of Anisotropic Hierarchical Structures Prepared by Femtosecond Laser 3D Printing and Their Applications in Crystallizing Microparticles. Acs Nano 9, 12060–12069 (2015). 26506428 10.1021/acsnano.5b04914

[b23] He Y. . “Overpass” at the junction of a crossed microchannel: An enabler for 3D microfluidic chips. Lab Chip 12, 3866–3869 (2012). 22871743 10.1039/c2lc40401j

[b24] Lim T. W. . Three-dimensionally crossing manifold micro-mixer for fast mixing in a short channel length. Lab Chip 11, 100–103 (2011). 20938497 10.1039/c005325m

[b25] Amato L. . Integrated three-dimensional filter separates nanoscale from microscale elements in a microfluidic chip. Lab Chip 12, 1135–1142 (2012). 22318474 10.1039/c2lc21116e

[b26] Wu D. . Ship-in-a-bottle femtosecond laser integration of optofluidic microlens arrays with center-pass units enabling coupling-free parallel cell counting with a 100% success rate. Lab Chip 15, 1515–1523 (2015). 25622687 10.1039/c4lc01439a

[b27] Xiong W. . Simultaneous additive and subtractive three-dimensional nanofabrication using integrated two-photon polymerization and multiphoton ablation. Light: Science & Applications 1, e6 (2012).

[b28] Hu Y. . High-efficiency fabrication of aspheric microlens arrays by holographic femtosecond laser-induced photopolymerization. Appl Phys Lett 103, 141112 (2013).

[b29] Zhang C. . A rapid two-photon fabrication of tube array using an annular Fresnel lens. Opt Express 22, 3983–3990, 10.1364/Oe.22.003983 (2014). 24663719

[b30] Zhang S. . Two-photon polymerization of a three dimensional structure using beams with orbital angular momentum. Appl Phys Lett 105, 061101 (2014).

[b31] Yang L. . Two-photon polymerization of cylinder microstructures by femtosecond Bessel beams. Appl Phys Lett 105, 041110 (2014).

[b32] Mills B., Grant-Jacob J. A., Feinaeugle M. & Eason R. W. Single-pulse multiphoton polymerization of complex structures using a digital multimirror device. Opt Express 21, 14853–14858 (2013). 23787672 10.1364/OE.21.014853

[b33] Yang L. . Projection two-photon polymerization using a spatial light modulator. Opt Commun 331, 82–86 (2014).

[b34] Aagedal H., Schmid M., Teiwes S. & Wyrowski F. Theory of speckles in diffractive optics and its application to beam shaping. J Mod Optic 43, 1409–1421, 10.1080/095003496155283 (1996).

[b35] Senthilkumaran P., Wyrowski F. & Schimmel H. Vortex Stagnation problem in iterative Fourier transform algorithms. Opt Laser Eng 43, 43–56, 10.1016/j.optlaseng.2004.06.002 (2005).

[b36] Golan L. & Shoham S. Speckle elimination using shift-averaging in high-rate holographic projection. Opt Express 17, 1330–1339 (2009). 19188961 10.1364/oe.17.001330

[b37] Pasienski M. & DeMarco B. A high-accuracy algorithm for designing arbitrary holographic atom traps. Opt Express 16, 2176–2190, 10.1364/Oe.16.002176 (2008). 18542298

[b38] Kotlyar V. V., Seraphimovich P. G. & Soifer V. A. An iterative algorithm for designing diffractive optical elements with regularization. Opt Laser Eng 29, 261–268, 10.1016/S0143-8166(97)00114-0 (1998).

[b39] Ripoll O., Kettunen V. & Herzig H. P. Review of iterative Fourier-transform algorithms for beam shaping applications. Opt Eng 43, 2549–2556 (2004).

[b40] Ovsianikov A. . Ultra-low shrinkage hybrid photosensitive material for two-photon polymerization microfabrication. Acs Nano 2, 2257–2262 (2008). 19206391 10.1021/nn800451w

[b41] Di Carlo D., Wu L. Y. & Lee L. P. Dynamic single cell culture array. Lab Chip 6, 1445–1449 (2006). 17066168 10.1039/b605937f

[b42] Chen X., Shojaei-Zadeh S., Gilchrist M. L. & Maldarelli C. A lipobead microarray assembled by particle entrapment in a microfluidic obstacle course and used for the display of cell membrane receptors. Lab Chip 13, 3041–3060 (2013). 23748734 10.1039/c3lc50083g

[b43] Dura B., Liu Y. & Voldman J. Deformability-based microfluidic cell pairing and fusion. Lab Chip 14, 2783–2790 (2014). 24898933 10.1039/c4lc00303a

[b44] Skelley A. M., Kirak O., Suh H., Jaenisch R. & Voldman J. Microfluidic control of cell pairing and fusion. Nat Methods 6, 147–152 (2009). 19122668 10.1038/nmeth.1290 PMC3251011

[b45] Tang Y. . Microfluidic device with integrated microfilter of conical-shaped holes for high efficiency and high purity capture of circulating tumor cells. Sci Rep-Uk 4, 6052, 10.1038/srep06052, http://www.nature.com/articles/srep06052#supplementary-information (2014). PMC7365311 25116599

[b46] Hernandez O. . Three-dimensional spatiotemporal focusing of holographic patterns. Nat Commun 7 (2016). 10.1038/ncomms11928 PMC4912686 27306044

