# Supplementary material for: Optimized holographic femtosecond laser patterning method towards rapid integration of high-quality functional devices in microchannels
Source: Sci Rep. 2016 Sep 13;6:33281. doi: 10.1038/srep33281 (PMC5020409; doi:10.1038/srep33281)
Supplement: Supplementary Information [file srep33281-s1.pdf]

Supplementary Information

Optimized holographic femtosecond laser patterning method towards rapid integration of high-quality functional devices in microchannels

Chenchu Zhang[1], Yanlei Hu[1*], Wenqiang Du[1], Peichao Wu[1], Shenglong Rao[1], Ze Cai[1], Zhaoxin Lao[1], Bing Xu[1], Jincheng Ni[1], Jiawen Li[1], Gang Zhao[1], Dong Wu[1*], Jiaru Chu[1] and Koji Sugioka[2]

[1]CAS Key Laboratory of Mechanical Behavior and Design of Materials, Department of Precision Machinery and Precision Instrumentation, University of Science and Technology of China, Hefei 230026, China

[2]Laser Technology Laboratory, RIKEN, 2-1 Hirosawa, Wako, Saitama 351-0198, Japan

Email: huyl@ustc.edu.cn and dongwu@ustc.edu.cn

Table of Contents

- Figures S1 to S6 (pages 2 to 7)
- Captions for Supporting Videos 1 and 2 (page 8)

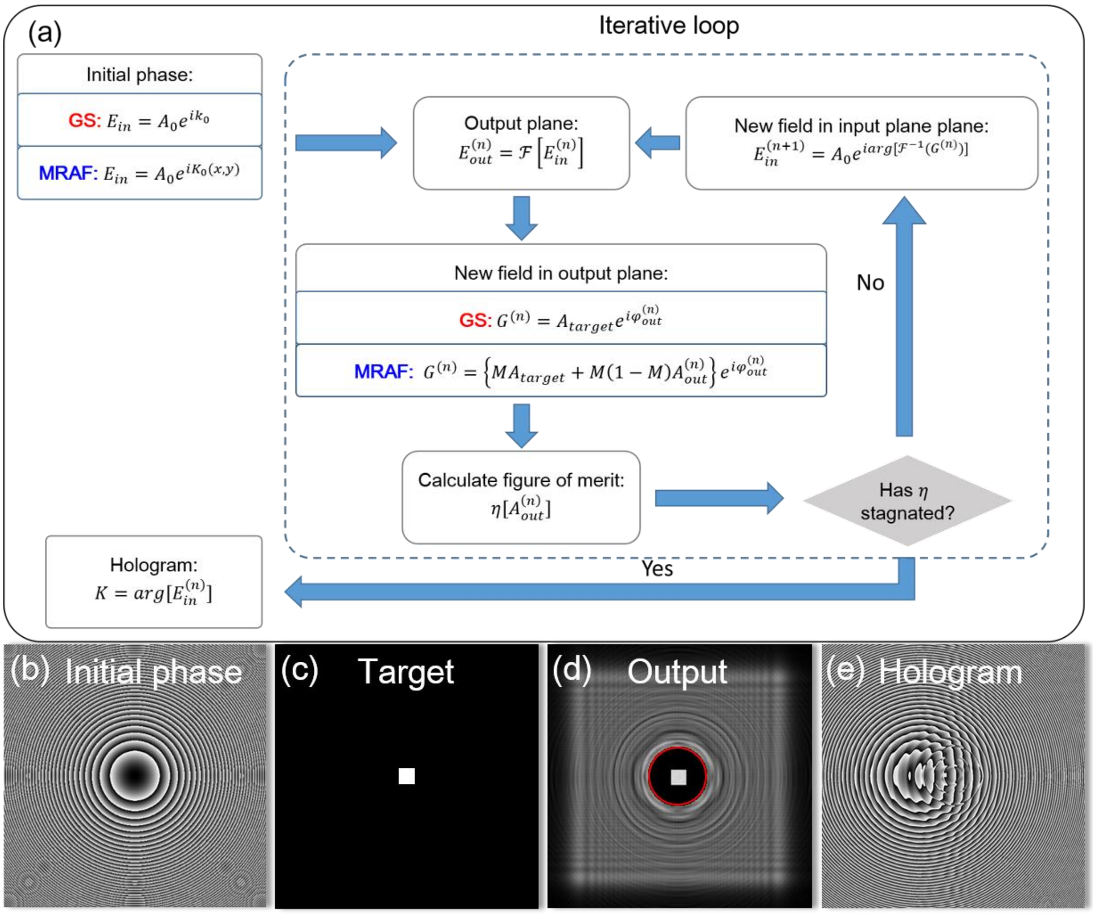

Figure S1. The calculation detail of MRAF algorithm.(a) is the iterative loop of MRAF algorithm and GS algorithm. In MRAF algorithm, the initial phase $K_0(x, y)$ is composed of linear and conical gradients to avoid introducing undesired optical vortices, as shown in (b). Then, the initial phase is fast Fourier transformed to the output plane. The intensity of the output plane is further replaced by the target shown in (c). It is a remarkable fact that in MRAF algorithm, only the intensity of "Signal Region" [within the red circle in (d)] is replaced, leaving "Noise Region" (out of the red circle in (d)) unchanged. After coding the Signal Region, a new output plane is inverse fast Fourier transformed to generate the new input plane and to start a new iteration. After tens of iteration, the merit of output plane remains stable. Then the output plane is exported to get the hologram [shown in (e)]. The calculation time of GS and MRAF algorithm is 35.24s and 36.73s. (100 iteration loops, Window 10 64-bit, Core I7-6700HQ, 8.00 GB RAM).

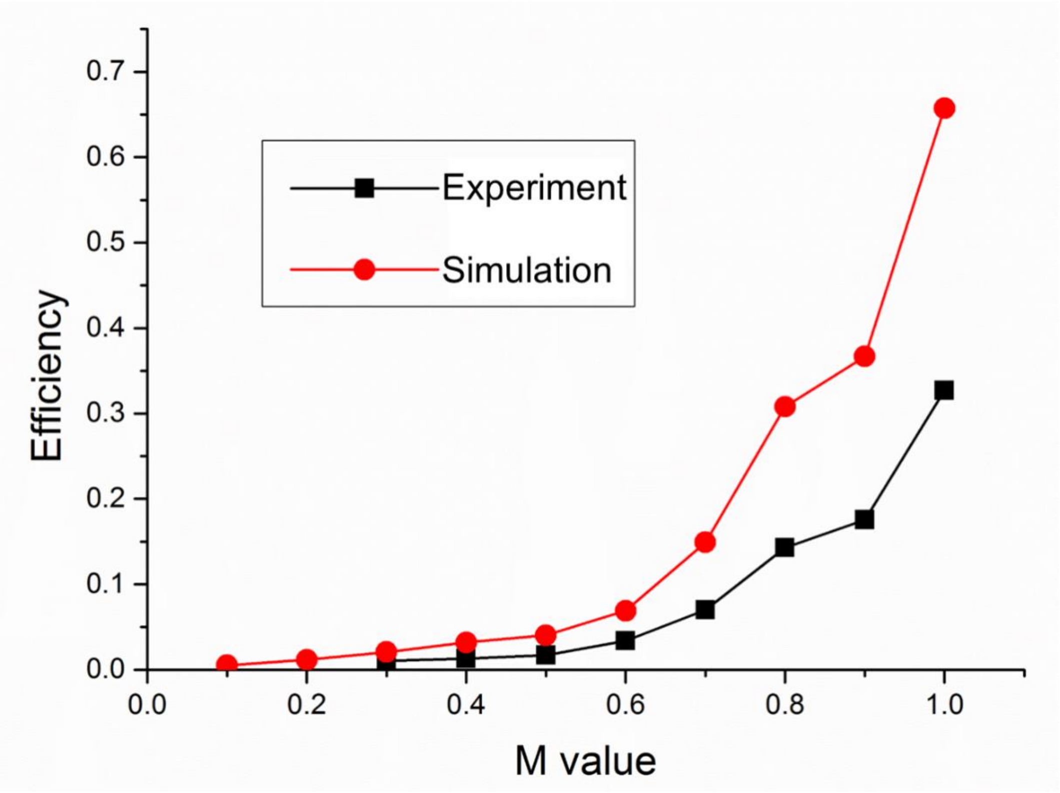

Figure S2. The diffraction efficiency of MRAF-CGHs with different M value. The experiment and simulation results show that the efficiency increases as the M value rises.

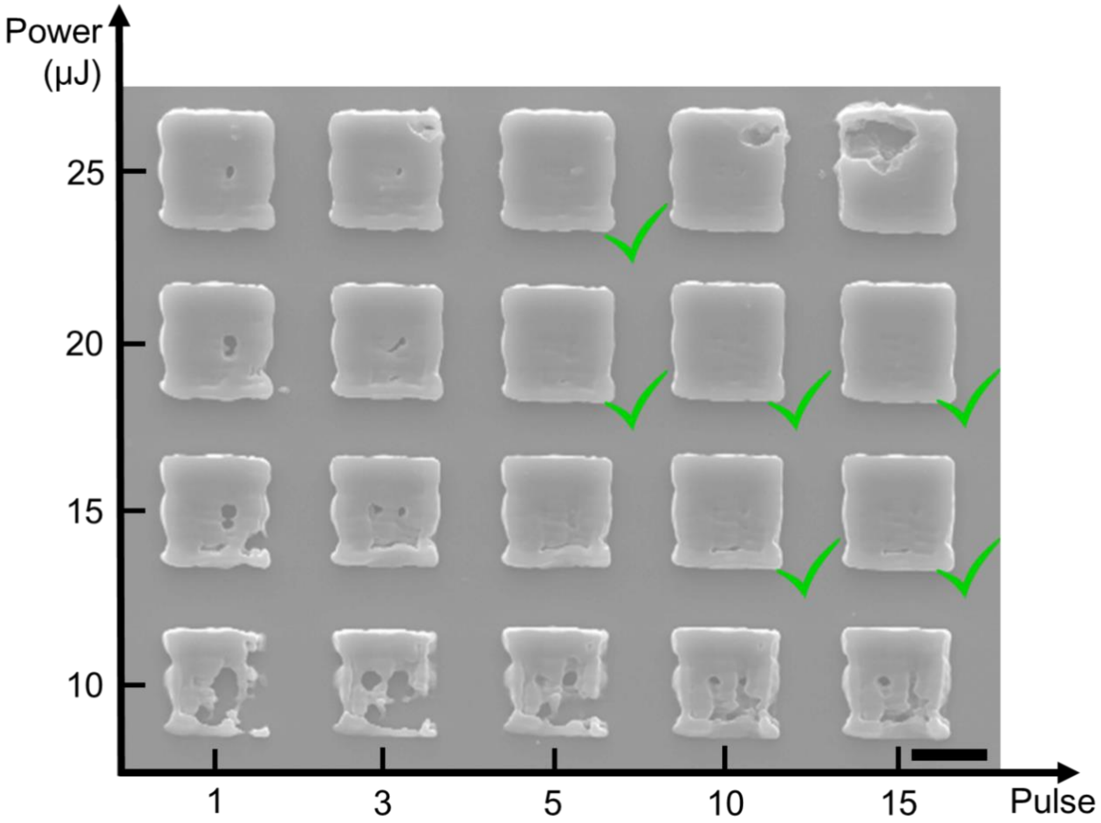

Figure S3. SEM images of rectangle array processed by different pulse numbers ranging from 1 to 15 and pulse power ranging from 10 to 25 μJ. The polymer is not fully polymerized when the pulse number and energy is low, which results in flaw of structures, shown at the bottom left of rectangle array. However, when the pulse number and power exceed the damage threshold of polymer (shown at the top right of array), the over exposed region is burned up, making a decline of surface quality. Scale bar: 10 μm.

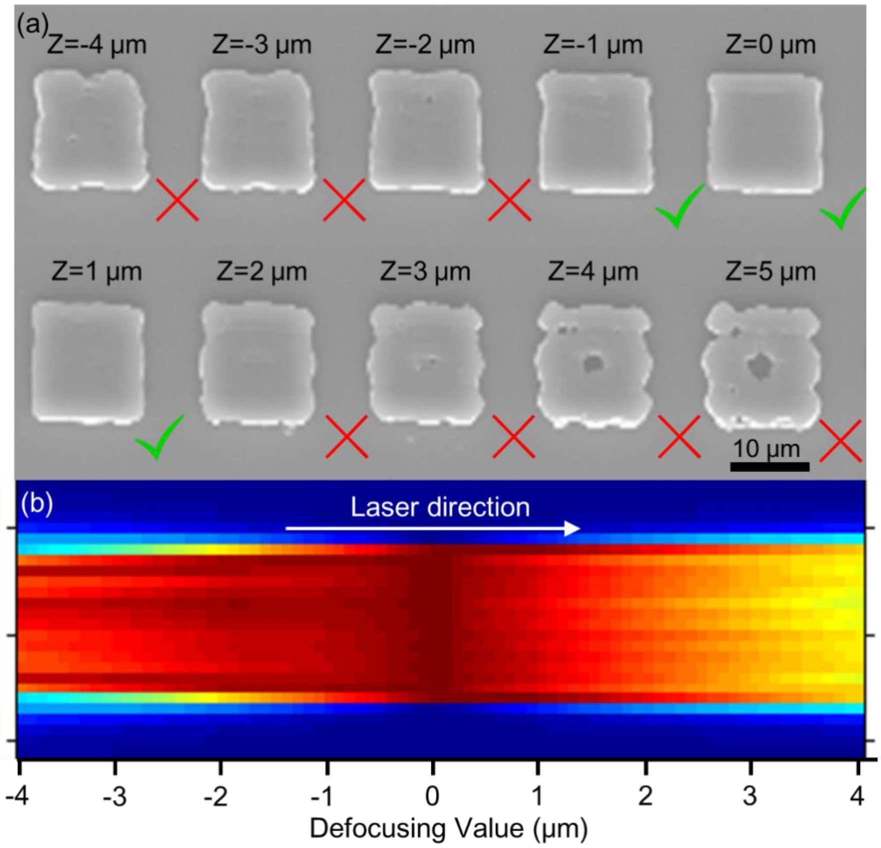

Figure S4. Morphology of micropatterns exposed at defocusing values varies from -4 µm to 5 µm. Each rectangle was fabricated with 20 µJ, 10 pulses. Structures fabricated with defocusing value at -1, 0, 1 µm have good surface quality, corresponding to the simulation in (b), which shows the intensity maintains high uniformity when the defocusing value varies from -1 to 1µm. Scale bar: 10 µm.

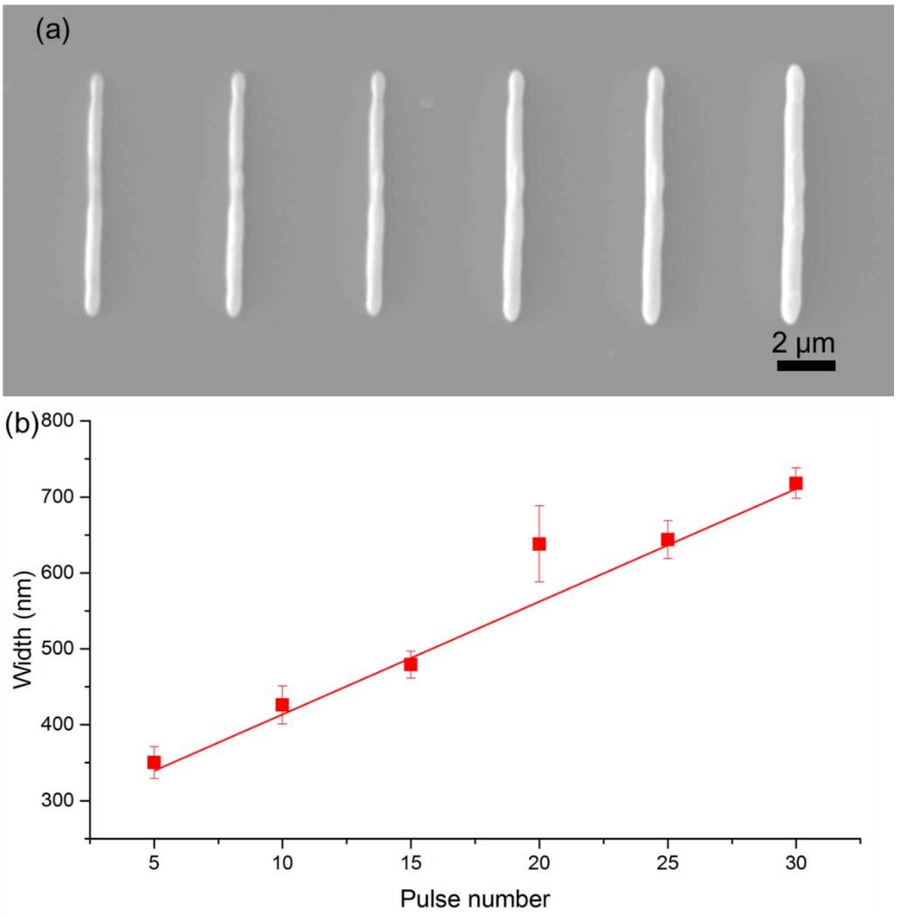

Figure S5. Resolution of single-pixel line processed by MHP. (a) is the SEM image of single-pixel line fabricated by the pulse number varies from 5 to 30. The pulse energy remains 15 μJ. The average width of each line is shown in (b), changing from 350 to 718 nm. The width of single-pixel line can be further reduced by lowering the pulse energy and less pulse number.

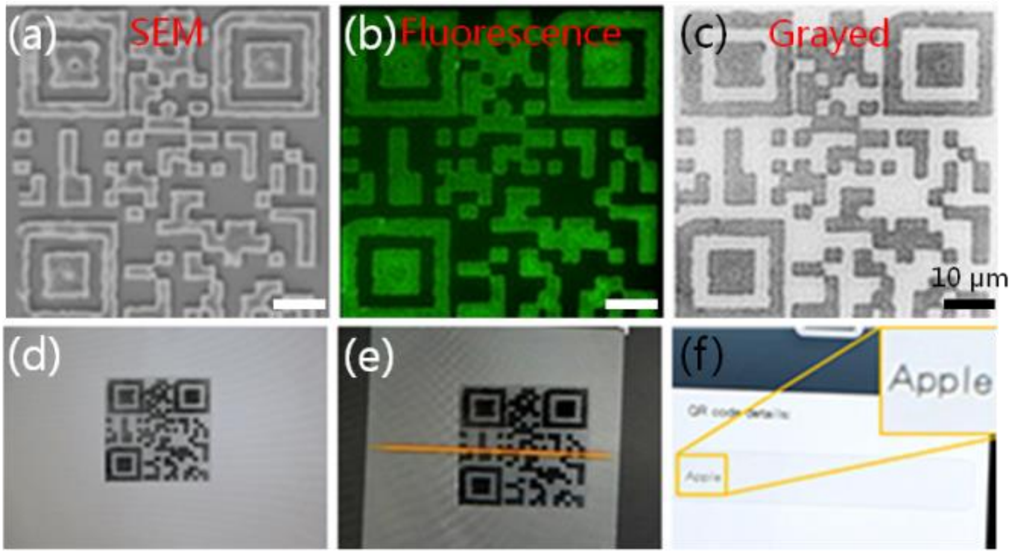

Figure S6. The detailed reading process of QR code.(a) is the SEM image of QR code containing information "Apple". The information of QR code can be highlighted by a fluorescence image, shown in (b). The fluorescence image is further grayed and reversed to be a standard QR code, which is shown in (c).The standard QR code is displayed on a computer screen for a QR scanner to read the information [(d)-(e)]. The information is retrieved in (f). Scale bars are 10 μm.

Supporting Video 1. The reading process of micro QR code. The grayed QR code displayed on a computer screen is read by a cell phone in less than 1 second.

Supporting Video 2. The capture function of microtrap array fabricated by MHP. The biggest particles are captured by the traps. Smaller particles can pass through the trap (a 3.0 μm bead in 18 s), moving around the trap (a 5.0 μm bead in 105 s) or moving around the blocked traps and passing through the unblocked traps (a 3.0 μm bead in 100 s). The flow speed is 1200 μm/s at the beginning and 200 μm/s at the end of the video, showing the trap array performs properly in a very wide flow speed range.
